# Severe central nervous system demyelination in Sanfilippo disease

**DOI:** 10.3389/fnmol.2023.1323449

**Published:** 2023-12-13

**Authors:** Mahsa Taherzadeh, Erjun Zhang, Irene Londono, Benjamin De Leener, Sophie Wang, Jonathan D. Cooper, Timothy E. Kennedy, Carlos R. Morales, Zesheng Chen, Gregory A. Lodygensky, Alexey V. Pshezhetsky

**Affiliations:** ^1^Department of Pediatrics, Centre Hospitalier Universitaire (CHU) Sainte-Justine Research Centre, University of Montreal, Montreal, QC, Canada; ^2^Department of Anatomy and Cell Biology, McGill University, Montreal, QC, Canada; ^3^NeuroPoly Lab, Institute of Biomedical Engineering, Department of Computer Engineering and Software Engineering, École Polytechnique de Montréal, Montreal, QC, Canada; ^4^Pediatric Storage Disorders Laboratory (PSDL), Departments of Pediatrics, Genetics and Neurology, Washington University School of Medicine, St. Louis, MO, United States; ^5^Department of Neurology and Neurosurgery, Montreal Neurological Institute, McGill University, Montreal, QC, Canada

**Keywords:** mucopolysaccharidosis, oligodendrocyte, myelination, lysosomal storage, GM3 ganglioside, diffusion basis spectrum imaging

## Abstract

**Introduction:**

Chronic progressive neuroinflammation is a hallmark of neurological lysosomal storage diseases, including mucopolysaccharidosis III (MPS III or Sanfilippo disease). Since neuroinflammation is linked to white matter tract pathology, we analyzed axonal myelination and white matter density in the mouse model of MPS IIIC *Hgsnat*^*P*304*L*^ and post-mortem brain samples of MPS III patients.

**Methods:**

Brain and spinal cord tissues of human MPS III patients, 6-month-old *Hgsnat*^*P*304*L*^ mice and age- and sex-matching wild type mice were analyzed by immunofluorescence to assess levels of myelin-associated proteins, primary and secondary storage materials, and levels of microgliosis. Corpus callosum (CC) region was studied by transmission electron microscopy to analyze axon myelination and morphology of oligodendrocytes and microglia. Mouse brains were analyzed *ex vivo* by high-filed MRI using Diffusion Basis Spectrum Imaging in Python-Diffusion tensor imaging algorithms.

**Results:**

Analyses of CC and spinal cord tissues by immunohistochemistry revealed substantially reduced levels of myelin-associated proteins including Myelin Basic Protein, Myelin Associated Glycoprotein, and Myelin Oligodendrocyte Glycoprotein. Furthermore, ultrastructural analyses revealed disruption of myelin sheath organization and reduced myelin thickness in the brains of MPS IIIC mice and human MPS IIIC patients compared to healthy controls. Oligodendrocytes (OLs) in the CC of MPS IIIC mice were scarce, while examination of the remaining cells revealed numerous enlarged lysosomes containing heparan sulfate, GM3 ganglioside or “zebra bodies” consistent with accumulation of lipids and myelin fragments. In addition, OLs contained swollen mitochondria with largely dissolved cristae, resembling those previously identified in the dysfunctional neurons of MPS IIIC mice. *Ex vivo* Diffusion Basis Spectrum Imaging revealed compelling signs of demyelination (26% increase in radial diffusivity) and tissue loss (76% increase in hindered diffusivity) in CC of MPS IIIC mice.

**Discussion:**

Our findings demonstrate an important role for white matter injury in the pathophysiology of MPS III. This study also defines specific parameters and brain regions for MRI analysis and suggests that it may become a crucial non-invasive method to evaluate disease progression and therapeutic response.

## Introduction

Two-thirds of patients diagnosed with Lysosomal Storage Diseases (LSDs), inherited metabolic disorders affecting lysosomal catabolism, show neurological symptoms and/or have pathological changes in the CNS (Boustany, 2013; Para et al., 2020). Two LSDs, Krabbe disease, caused by deficiency of β-galactocerebrosidase (GALC), and Metachromatic leukodystrophy (MLD), caused by deficiency of arylsulfatase A (ARSA), belong to the class of leukodystrophies, manifesting with white matter pathology and progressive degeneration of myelin sheaths (Maegawa, 2019). Progressive myelin defects in these disorders are caused by two specific sphingolipids accumulating in the brains of affected patients, galactosylceramide (psychosine) in the case of Krabbe disease and sulfatide in the case of MLD (Maegawa, 2019). Both galactosylceramide and sulfatide are important components of myelin sheaths and are generated by myelin-producing cells, oligodendrocytes (OLs) in CNS, and Schwann cells in the peripheral nervous system. However, when their levels are drastically increased, as a result of genetic deficiencies of GALC and ARSA, respectively, they become highly toxic for OLs and Schwann cells. Because of progressive myelin loss, both Krabbe and MLD patients, especially those having complete or almost complete deficiencies of the enzymes involved, manifest with a severe neurological impairment and deterioration leading to death before the age of 5 years (Wenger et al., 1997, 2000; Gieselmann, 2008). In both Krabbe disease and MLD, MRI brain imaging was especially instrumental for the identification and characterization of white matter defects (Groeschel et al., 2011; Poretti et al., 2014; Sabourdy et al., 2015).

Since glycosphingolipids are critical components of myelin sheaths (Kishimoto, 1986; de Vries and Hoekstra, 2000), demyelination and white matter pathology have been also reported in patients affected with lysosomal glycosphingolipidoses, including infantile forms of Niemann-Pick disease type C (NPC) (Maegawa, 2019). In the latter case, demyelination was proposed to result from the secondary storage of GM3 ganglioside in the lysosomes of OLs leading to their dysfunction (Lee et al., 2014).

Mucopolysaccharidosis type III (MPS III) or Sanfilippo disease, remains the most prevalent untreatable neurological lysosomal disorder (Heon-Roberts et al., 2020). MPS III is a spectrum of four conditions (MPS IIIA-D), caused by defects in the genes encoding the enzymes involved in lysosomal degradation of a glucosamine, heparan sulfate (HS). In MPS III, HS accumulates in brain tissue and causes neuronal dysfunction and death leading to neuropsychiatric problems, developmental delays, childhood dementia, blindness and death during the second decade of life (Heon-Roberts et al., 2020).

Previous studies involving MPS III patients and animal models revealed that disrupted catabolism of HS in the brain causes multifaceted pathological response, from neuroinflammation and oxidative stress to impairment of autophagy, neuronal accumulation of misfolded proteins, GM2 or GM3 gangliosides, and synaptic defects. Together, these processes contribute to neuronal dysfunction and neurodegeneration which lead to cognitive and motor decline in patients (Heon-Roberts et al., 2020). Importantly, in most Sanfilippo patients the rate of progressive functional impairment correlates with that of cortical and cerebellar atrophy, ventricular volume increase and other brain abnormalities, detected by computed tomography-scan or MRI, suggesting that these symptoms are associated with neuronal loss and pathological gray matter lesions (Shapiro et al., 2016; Whitley et al., 2018). Much less is known, however, about the white matter defects and, in particular, axonal pathology and demyelination in the MPS III patients. While some case reports have indicated the presence of diffuse high-intensity signal in the white matter of MPS IIIA and IIIB patients (Zafeiriou et al., 2001; Kara et al., 2008), other studies reported an absence of white-matter lesions in most MPS III cases (Reichert et al., 2021).

Here, for the first time, we report that progressive severe demyelination is a hallmark of CNS pathology in, both, human MPS IIIC patients and the mouse *Hgsnat*^*P*304*L*^ model (Pan et al., 2022) of the disease. We also demonstrate that, in the brains of MPS IIIC mice, the disruption of myelination results from reduced OL numbers and substantial pathological changes in OL morphology.

## Materials and methods

### Animals

The knock-in mouse model of MPS IIIC, *Hgsnat*^*P*304*L*^, expressing the HGSNAT enzyme with human missense mutation P304L, on a C57BL/6J genetic background, has been previously described (Pan et al., 2022).

Mouse studies were performed in accordance with the Canadian Council on Animal Care (CCAC) in the CCAC-accredited animal facility of the CHU Ste-Justine (protocol 2022-3453). All mice were housed in an enriched environment in poly-carbonate cages under 12:12 h light: dark cycles in a temperature- and humidity-controlled room. Mice had access to a normal rodent chow and water *ad libitum*. The animals were bred as homozygous couples for both WT and *Hgsnat*^*P*304*L*^ strains. Experiments were conducted using both male and female mice and the data analyzed to identify potential differences between sexes. Since no differences between sexes were observed in the experiments, the data for males and females were combined. Wild-type siblings were used as controls.

### Analysis of human brain tissues

Post-mortem brain tissues from two patients with MPS IIIC, fixed in 10% buffered formalin, were collected for neuropathological examination. The first brain was provided by the Anatomic Gift Registry while the second brain was donated for research purposes by the parents of the patient. Brain tissues of age-matched patients without CNS pathology were provided, together with clinical descriptions, by the Neuromax biobank of CHU Sainte-Justine.

The brains underwent gross examination by a neuropathologist (ZC), and representative regions were sampled and processed for paraffin embedding. Four-μm sections were cut from the formalin fixed paraffin embedded (FFPE) blocks, stained with Hematoxylin-Eosin (H&E), Saffron (HES), periodic acid-Schiff stain (PAS) and Luxol Fast Blue (LFB), and examined under the light microscope.

In addition, frozen or paraformaldehyde (PFA) fixed cerebral cortices from MPS patients (1 case of MPS IIIA, 1 case of MPS IIIC and 1 case of MPS IIID) and age-matched controls with no pathological changes in the CNS were provided by NIH NeuroBioBank (project 1071, MPS Synapse). Upon arrival to the laboratory, the samples were embedded in Tissue-Tek^®^ optimum cutting temperature (OCT, Sakura, USA) compound and stored at −80°C. A total of 40 μm thick brain sections were cut and stored in cryopreservation buffer (0.05 M sodium phosphate buffer pH 7.4, 15% sucrose, 40% ethylene glycol) and stored at −20°C until immunohistochemical labeling.

### Immunohistochemistry

Mice were perfused with 4% PFA in PBS. Following perfusion, brains were isolated and post-fixed in 4% PFA in PBS overnight. Brains were further incubated in 30% sucrose for 48 h at 4°C, embedded in OCT, cut into sequential 40 μm-thick coronal cross-sections using a Cryostat Epredia CryoStar NX50, and then kept at −20°C in cryopreservation buffer (0.05 M sodium phosphate buffer pH 7.4, 15% sucrose, 40% ethylene glycol). For immunofluorescence analysis, brain slices were permeabilized and blocked with 5% bovine serum albumin (BSA), and 0.3% Triton X-100 in PBS for 2 h at room temperature. Sections were then incubated with primary antibodies, diluted with 1% BSA and 0.1% Triton X-100 in PBS at 4°C overnight. The antibodies and their dilutions are listed in the Supplementary Table 1.

Mouse brain sections were washed with PBS and counterstained with Alexa Fluor^®^ 488-conjugated goat anti-mouse IgG (A21202), Alexa Fluor^®^ 555-conjugated goat anti-rabbit IgG (A21428), and Alexa Fluor^®^ 633-conjugated goat anti-mouse IgG (A21094) (dilution 1:400, all from Thermo Fisher Scientific) for 2 h at room temperature. To quench autofluorescence, the mouse brain sections were dipped briefly in TrueBlack^®^ Lipofuscin Autofluorescence Quencher (dilution 1:10, Biotium, 23007) for 1 min, and then washed with PBS. The slides were mounted with Prolong Gold Antifade mounting reagent with DAPI (Invitrogen, P36935) and captured using a Laser scanning confocal microscope (Leica TCS SP8: 20x, 40x, and 63x oil objective, N.A. 1.4). Z-stack projections were conducted with a step size of 0.3 μm to represent images.

For the analysis of spinal cord, adjacent one-in-forty-eight series of 40 μm coronal sections from each mouse were stained on slides using a modified immunofluorescence protocol (Nelvagal et al., 2021, 2022). Sections were labeled with rat anti-CD68 (1:400, Bio-Rad MCA1957) or rat anti-MBP (1:500, Merck Millipore, MAB386) primary antibodies followed by AlexaFluor546 goat anti-rat (1:500, Invitrogen A11081) for CD68, or AlexaFluor488 goat anti-rat (1:500, Invitrogen A48262) for MBP. Slides were counterstained with TrueBlack Lipofuscin Autofluorescence Quencher (Biotium).

To quantify microgliosis (CD68-positive activated microglia) and myelination (MBP immunoreactivity), semiautomated thresholding image analysis was performed as described previously (Nelvagal et al., 2021, 2022). This involved collecting slide-scanned images at 10x magnification (Zeiss Axio Scan Z1 Fluorescence Slide Scanner) from each animal. Contours of appropriate anatomical regions were then drawn, and images were subsequently analyzed with *Image-Pro Premier* (Media Cybernetics) using an appropriate threshold that selected the foreground immunoreactivity above the background. All thresholding data (CD68 and MBP) were expressed as the percentage of area within each anatomically defined region of interest that contained immunoreactivity above the set threshold for that antigen (“% immunoreactivity”).

### Image processing and analysis

Unless indicated otherwise, images were processed and quantified using ImageJ 1.50i (National Institutes of Health, Bethesda, MD, USA). Quantification was performed in a double-blind fashion. Three-dimensional images were generated using Imaris (Oxford Instruments, version 9.6) software (Bitplane).

### Immunoblotting

Half-brain sections from 6-month-old mice were homogenized in a non-denaturing lysis buffer (50 mM Tris–HCl pH 7.4, 150 mM NaCl, 1% NP-40, 0.25% sodium deoxycholate, 0.1% SDS, 2 mM EDTA, 1 mM PMSF), supplemented with protease and phosphate inhibitor cocktails (Sigma-Aldrich cat# 4693132001 and 4906837001). The homogenates were cleared by centrifugation at 13,000 × *g* at 4°C for 25 min, and the protein concentration in the collected supernatant was measured using a Pierce BCA Protein Assay Kit (Thermo Fisher Scientific). Protein extracts (20 μg of protein for each sample), were incubated in a boiling bath for 10 min and analyzed by SDS-PAGE on 4–20% precast polyacrylamide gradient gel (Bio-Rad, 4561096). Western blot analyses were performed according to standard protocols using antibodies against MBP (1:1000, Abcam, cat# ab218011), MAG (1:1000, Abcam, cat# ab89780), and α-tubulin (1:2000, DSHB, cat# 12G10) as a control. The immunoblots were revealed by chemiluminescence with SuperSignalWest Pico PLUS (Thermo Fisher Scientific, Waltham, MA, USA). Detected bands were quantified using ImageJ 1.50i software (National Institutes of Health, Bethesda, MD, USA) and normalized for the intensity of the α-tubulin band.

### Transmission electron microscopy analysis

To prepare sections for Transmission Electron Microscopy (TEM) analysis, 3 mice from each genotype were anesthetized with sodium pentobarbital (50 mg/kg BW) and perfused with phosphate-buffered saline (PBS, pH 7.4), followed by 2% paraformaldehyde/2.5% glutaraldehyde in 0.1 M sodium cacodylate buffer, pH 7.4. The brains were incubated in the same fixative for 24 h at 4°C, washed with MilliQ H2O, and post-fixed with 1% osmium tetroxide, 1.5% potassium ferrocyanide in H2O for 2 h, at 4°C. The samples were further dehydrated in a graded bath of acetone and MilliQ H2O with increasing concentrations of 30, 50, 70, 80, 90, and 3 × 100% for 8 min. The samples were infiltrated with 100% Epon, embedded in a rubber embedding mold, and polymerized in the oven at 60°C for 48 h. Once the resin was polymerized, semi-thin (1.0 μm) sections of the corpus callosum were dissected and stained with 1% toluidine blue before being mounted on glass slides and examined with a Leica DMS light microscope to select regions of interest. The sections were further cut into ultrathin (70–80 nm) sections, placed on 200 Mesh copper grids, stained with lead citrate, and examined at 80 kV on an FEI Tecnai G2 Spirit (FEI Company, Hillsboro, OR, USA) electron microscope equipped with a Morada CCD digital camera (Olympus, Tokyo, Japan). Micrographs were taken with 2900× and 4800× magnification.

To quantify the number of myelinated axons, a minimum of 10 images were analyzed for each animal. All myelinated axons within the counting frame were counted and included in the statistical analysis. Then, for each micrograph, 5 randomly selected axons were used to analyze axonal parameters including axonal diameter, myelin thickness and g-ratio, using ImageJ software.

### Preclinical high-field MRI

Two groups of 7-month-old mice (10 WT and 8 MPS IIIC) were imaged using a preclinical scanner at the Cerebral Imaging Centre of the Douglas Mental Health University Institute (Montreal, QC, Canada). Animals were perfused with PBS followed by 4% PFA in PBS, under terminal anesthesia, and brains were carefully removed and immersed in 4% PFA in PBS for 5 h. Brains were then mounted in a syringe with Fomblin oil for *ex vivo* MR imaging, using a solenoid coil custom-built to fit the syringe.

Imaging was carried out on a 7T Bruker MRI scanner. 2D spin-echo sequences with pulsed gradients were used to acquire diffusion-weighted data. Imaging included 1 b0 and 25 *b*-values (0 < b ≤ 3000 s/mm^2^) with different directions for each *b*-value, a field of view 12 mm × 12 mm, resolution, 0.15 mm × 0.15 mm × 0.4 mm, and TR/TE: 3300 ms/32 ms. Diffusion basis spectrum imaging (DBSI) and Diffusion imaging in Python-Diffusion tensor imaging (DIPY-DTI, non-linear algorithm) were used for reconstruction as described (Wang et al., 2011; Garyfallidis et al., 2014). On two consecutive slices, regions of interest (ROI) were manually selected on corpus callosum in color-coded fractional anisotropy (FA) maps extracted from DTI.

### Statistical analysis

Statistical analyses were performed using Prism GraphPad 9.3.0 software (GraphPad Software San Diego, CA, USA). The normality for all data was checked using the D’Agostino and Pearson omnibus normality test. Significance of the difference was determined using a Student’s *t*-test, when comparing two groups, and one-way ANOVA test followed by Tukey’s multiple comparison test, when comparing more than two groups. Two-way ANOVA followed by Bonferroni’s or Tukey’s *post-hoc* tests was used for two-factor analysis. The Mann-Whitney test was used on metrics extracted from DBSI when normality was not attainted. A *P*-value less than 0.05 was considered significant.

## Results

### Massive reduction of myelin-associated proteins in MPS IIIC mice

Chronic progressive neuroinflammation and microgliosis (increased brain levels of proinflammatory activated microglia) are well documented in lysosomal storage diseases including MPS IIIC. As chronic neuroinflammation and microglial activation can alter white matter tracts and interrupt communication between neurons (Han et al., 2019), we hypothesized that pathological changes in axon tracts might include a loss of myelin with decreased white matter density and contribute to CNS pathology in MPS IIIC patients. To investigate this hypothesis, we examined the integrity of myelination in our recently described *Hgsnat*^*P*304*L*^ mouse model expressing the mutant HGSNAT Pro304Leu variant (Pan et al., 2022). These mice reveal progressive pathological alterations in the cortical and subcortical gray matter, including pronounced synaptic defects, astromicrogliosis and neurodegeneration (Pan et al., 2022).

**FIGURE 1:**
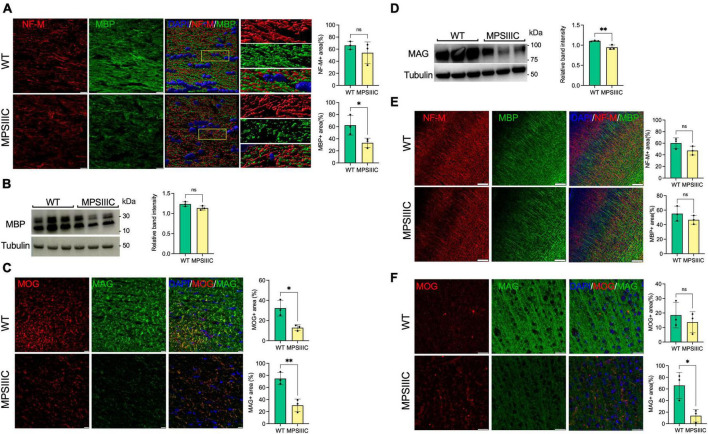
Myelin-associated proteins MBP, MAG, and MOG are reduced in the CC of MPS IIIC compared with WT mice, suggesting myelin loss. **(A)** Panels show representative images of the CC of 6-month-old WT and MPS IIIC mice (left) and three-dimensional (3D) enlarged images of the areas marked by yellow boxes (right). MBP + areas (green) on the surface of NF-M + axons (red) are reduced. The graphs show quantification of MBP + and NF-M + areas by ImageJ software. **(B)** Immunoblotting shows trend for reduction of MBP in the total brain homogenates of MPS IIIC mice. **(C)** IHC analysis reveals a reduction of MAG and MOG in CC of MPS IIIC mice compared with WT mice. Graphs show quantification of MAG + and MOG + stained area by ImageJ software. **(D)** Western blots of total protein extracts from brains of 6-month-old MPSIIIC mice confirm reduction of MAG. The graph shows intensities of MAG immunoreactive bands, quantified with ImageJ software and normalized by the intensity of tubulin immunoreactive bands. **(E)** No significant differences in MBP labeling in the cortex are detected by IHC between MPS IIIC and WT mice. **(F)** Level of MAG (green) labeling is reduced in the cortex of MPS IIIC compared with WT mice, while MOG (red) labeling shows only a non-significant trend for decrease. In all panels DAPI (blue) was used as a nuclear counterstain and scale bars equal 10 μm. All graphs show individual data, means and SD obtained for three mice per genotype. *P*-values were calculated using an unpaired *t*-test (*P < 0.05; **P < 0.01, ns, non-significant).

To assess for the presence of myelination defects, we first examined the levels of myelin-associated proteins, Myelin Basic Protein (MBP), Myelin Associated Glycoprotein (MAG) and Myelin Oligodendrocyte Glycoprotein (MOG), in the somatosensory cortices (SSC) and corpus callosum (CC) by immunohistochemical analysis of brain sections of 6-month-old *Hgsnat*^*P*304*L*^ mice compared with WT mice matched for age, sex, and genetic background. The immunoreactivity detected for each protein was significantly reduced in the CC of MPS IIIC mice at 6 months of age compared with WT counterparts (Figures 1A–C). In SSC, levels of MAG were significantly reduced, while non-significant trends toward a decrease were observed for MBP and MOG (Figures 1D–F). The axonal marker, Neurofilament medium chain protein (NF-M), also showed a non-significant trend toward a decrease in both areas. Together, these results were consistent with white matter injury in the brain of 6-month-old *Hgsnat*^*P*304*L*^ mice.

**FIGURE 2:**
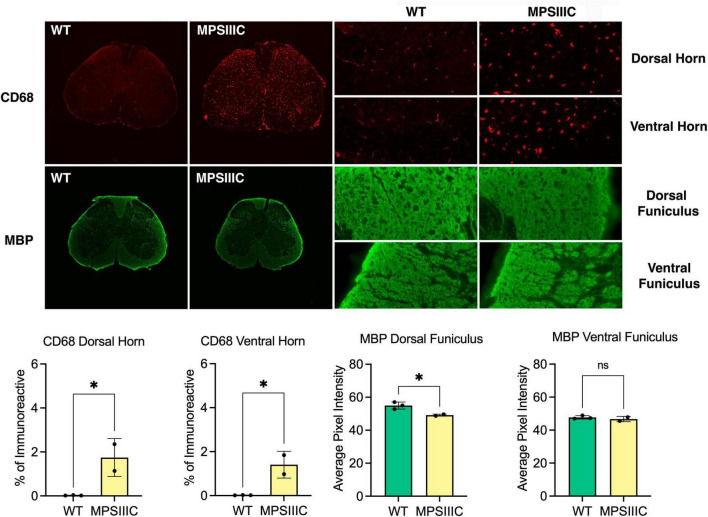
Microglial activation and white matter changes in the lumbar spinal cord of 6-month-old MPS IIIC mice. Representative images of immunostaining for the microglial marker CD68 (red) and myelin basic protein (MBP green) in the lumbar cord of 6-month-old *Hgsnat*^*P*304*L*^ (MPS IIIC) mice and age-matched WT mice. Numerous CD68 immunoreactive microglia with enlarged cell soma are present in the gray and white matter of the dorsal and ventral horn of MPS IIIC mice but are virtually absent in WT mice at this age. Compared to age-matched WT mice, MBP immunoreactivity is moderately reduced in the dorsal funiculus of 6-month-old MPS IIIC mice, but unchanged in the ventral funiculus. Quantitative thresholding image analysis confirmed these observations revealing significantly elevated CD68 immunoreactivity in the dorsal and ventral horn of 6-month-old MPS IIIC vs. age-matched WT mice. Similarly, there was significantly less MBP immunoreactivity in the dorsal funiculus of 6-month-old MPS IIIC vs. age-matched WT mice, but no significant change in the ventral funiculus. All graphs show individual data, means and SD obtained for two mice per genotype. *P*-values were calculated using an unpaired *t*-test (*P < 0.05; ns, non-significant).

In the spinal cord of 6-month-old MPS IIIC mice, immunostaining for the microglial marker CD68 revealed numerous intensely stained microglia with enlarged cell soma in the gray and white matter, while age matched WT mice revealed only few lightly stained microglia with a small cell soma (Figure 2). Immunostaining for MBP revealed intense immunoreactivity within the spinal white matter of mice of both genotypes. Thresholding image analysis confirmed that significantly more CD68 immunoreactivity was present in both the dorsal and ventral gray matter of MPS IIIC mice. We detected a moderate yet significant reduction in the intensity of MBP immunoreactivity in the dorsal funiculus of MPS IIIC mice, but no significant difference between genotypes in MBP immunoreactivity in the ventral funiculus (Figure 2).

### Reduction of myelin thickness and axon degeneration in the CC of MPS IIIC mice

**FIGURE 3:**
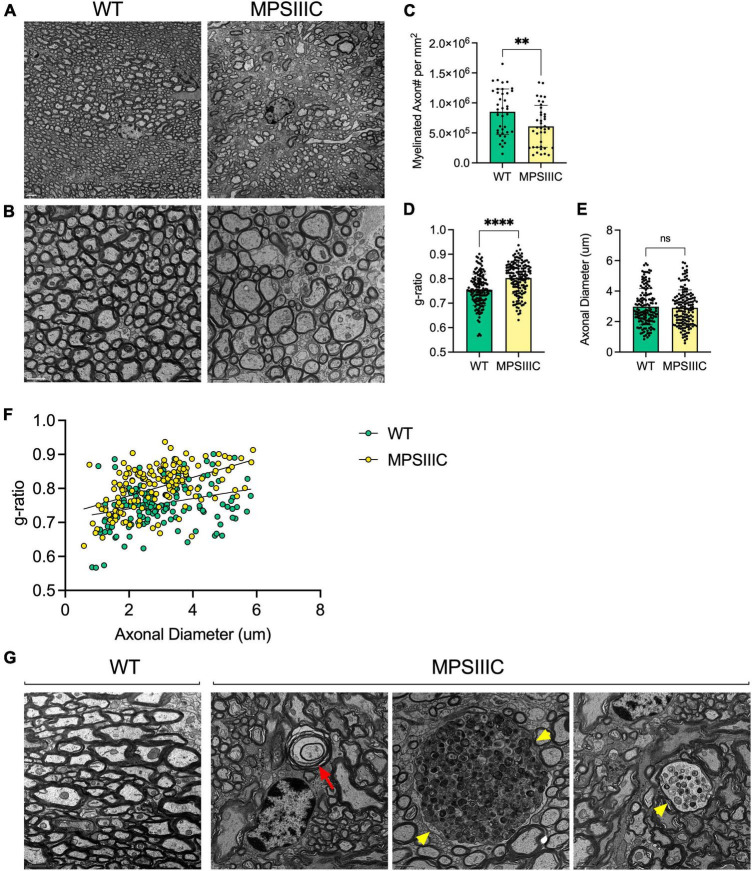
Transmission electron microscopy reveals reduced myelin thickness, decreased number of myelinated axons, structural defects in myelin sheaths and axonal swelling in the corpus callosum of MPS IIIC mouse. Panels **(A,B)** show representative TEM images of CC of 6-month-old MPS IIIC and WT mice taken at lower (2000X) and higher (4000X) magnifications, respectively. Scale bars equal 2 mm **(A)** and 1 mm **(B)**. **(C)** The graph shows quantification of myelinated axon density (mean number of myelinated axons per square millimeter) in CC of WT and MPS IIIC mice. **(D)** G-ratio values for myelinated axons in CC of MPS IIIC mice are significantly higher than those for axons of WT mice. **(E)** Axonal diameters are similar in WT and MPS IIIC mice. **(F)** Scatter plot depicting g-ratio vs. axonal diameter values. Graphs in panels **(D,E)** show individual values, means and SD and in the panel **(F)** individual values and linear regression plots. Sections from 3 mice per genotype (50 randomly selected axons per mouse) were analyzed. *P* values were calculated using *t*-test; **P < 0.01; ****P < 0.0001; and ns, non significant. **(G)** Electron micrographs show an absence of pathological changes in the axons of WT mice. In contrast, degenerated axons with empty myelin sheath and split myelin (red arrow), as well as large axonal swellings containing accumulating vesicles (yellow arrowheads), are observed in MPS IIIC mice.

To determine if changes in the amounts of myelin associated proteins in the CC coincided with alterations in myelin structure, three MPS IIIC and three age/sex matched WT mice were examined at the ultrastructural level by TEM followed by quantification of axon diameters and myelin thickness. We detected a relative scarcity of myelinated axonal profiles in MPS IIIC mice, and the axons that were myelinated had an average g-ratio (axon diameter/myelinated fiber diameter) of 0.803 ± 0.134, that was significantly higher than the one for WT mice, 0.76 ± 0.15, indicating a reduced thicknesses of myelin sheaths (Figure 3). Scatter plots of g-ratios vs. axonal diameter indicated hypomyelination of axons of all sizes in MPS IIIC mice. At the same time, no difference in the mean axonal diameters was detected between WT and MPS IIIC mice. Notably, in control animals, myelin thickness showed the expected positive correlation with axon diameter (*R*^2^ = 0.25), while, in MPS IIIC mice, myelin thickness did not significantly correlate with axon diameter (*R*^2^ = 0.08). TEM examination revealed defects in myelination along axons in CC (empty myelin sheath and splits in the compact myelin) with axonal swelling (spheroids) containing accumulated storage and/or transport vesicles. Similar swellings that coincided with microtubule defects were previously reported by us in hippocampal CA1 pyramidal neurons and in cultured neurons of another MPS IIIC model (*Hgsnat-Geo* mice) along with the evidence that the swellings disrupt synaptic vesicle precursor transport along axons (Para et al., 2021).

### Activated microglia in CC of MPS IIIC mice accumulate myelin debris

**FIGURE 4:**
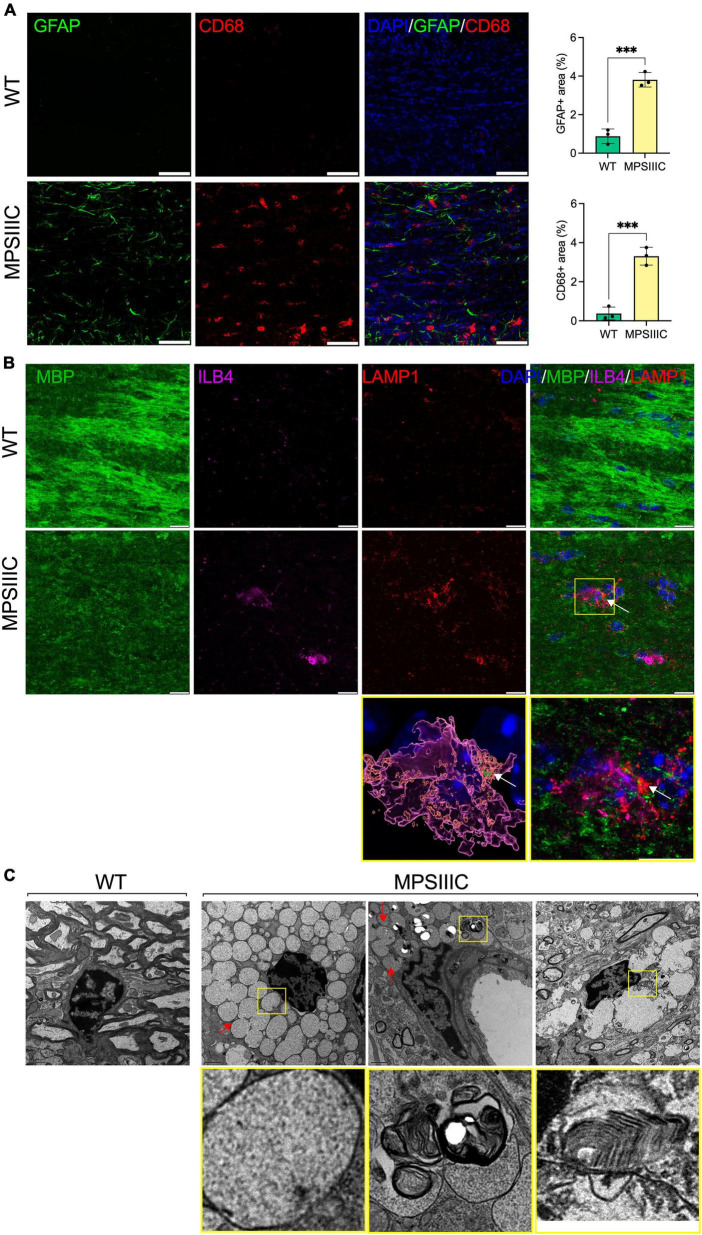
Microglia in CC of MPS IIIC but not of WT mice show lysosomal accumulation of myelin fragments and GAGs. **(A)** Panels show representative confocal microscopy images of CC tissue of 6-month-old WT and MPS IIIC mice labeled with antibodies against GFAP (green) and CD68 (red), markers for astrocytes and activated microglia, respectively. DAPI (blue) was used as a nuclear counterstain. Scale bar equals 50 μm. Graph shows quantification of GFAP + and CD68 + areas. Individual values, means and SD (*n* = 3) are shown. *P* values were calculated using *t*-test; ***P < 0.001. **(B)** Panels show representative confocal microscopy images of the CC of 6-month-old MPS IIIC and WT mice labeled with fluorescent isolectin b4 (ILB4, purple), and antibodies against MBP (green), and LAMP1 (red). DAPI (blue) was used as a nuclear counterstain. Scale bars equal 10 μm. In the yellow box on the right, the enlarged confocal image of the boxed area shows the colocalization of MBP + puncta with LAMP1 + lysosomal marker inside ILB4 + activated microglia in the CC of a MPSIIIC mouse. On the left, 3D reconstruction shows that MBP + puncta (arrows) are located inside the LAMP1 + lysosome of a microglia cell. **(C)** Both electron-lucent vacuoles (arrow), consistent with HS storage, and those containing “zebra bodies,” indicative of myelin debris (boxed), are detected in microglia in the CC of MPS IIIC mouse. Microglia in the CC of WT mouse are small and do not contain storage vacuoles. Scale bar equals 1 μm.

Microglia phagocytose myelin sheaths to modify myelination and preserve its integrity and function (Safaiyan et al., 2016; Thériault and Rivest, 2016). To verify if this process is altered in the brain tissues of MPS IIIC mice, we analyzed them using IHC and detected MBP-positive puncta in ILB4-positive (Figure 4A) and CD68-positive (Supplementary Figure 1) microglia in the CC. This puncta was not present in the tissues of WT mice. Moreover, MBP-positive puncta in microglia localized inside enlarged LAMP1-positive vacuoles, confirming lysosomal accumulation of MBP fragments in these cells (Figure 4B). The IHC results were confirmed by TEM examination revealing that microglia (Savage et al., 2018) in the CC of MPS IIIC mice were enlarged and contained vacuoles with so called “zebra bodies,” consistent with myelin accumulation (Pagni et al., 2012; Figure 4C, boxed). We also detected electrolucent vacuoles most probably containing HS and other glycosaminoglycans (Figure 4C, arrows; Martins et al., 2015). In the WT mice, the microglia remained small and did not contain enlarged vacuoles (Figure 4C). Notably, CD68-positive activated microglia were detected in MPS IIIC brain as early as P25, when the levels of MBP in the CC are still intact (Supplementary Figure 2), suggesting that demyelination was likely not caused by direct action of the immune cells.

### Oligodendrocyte dysfunction in MPS IIIC mice

**FIGURE 5:**
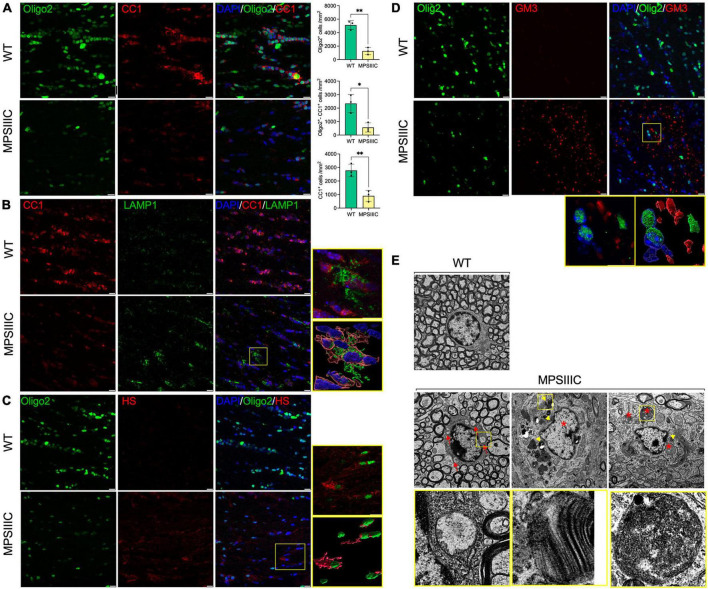
Oligodendrocytes in CC of MPS IIIC mice show reduced abundance, maturation and morphological abnormalities. **(A)** Representative confocal microscopy images of CC of 6-month-old WT and MPS IIIC mice immunolabelled for OL lineage marker Olig2 (green) and mature OL marker CC1 (red). Graphs show quantification of Oligo2 + , CC1 + and Oligo2 + /CC1 + cells (the number of cells per mm^2^) in WT and MPS IIIC mice. Individual data, means and SD (*n* = 3) are shown. *P* values were calculated using *t*-test; *P < 0.05 and **P < 0.01. **(B)** Images of CC of 6-month-old WT and MPS IIIC mice immunolabelled for CC1 (red) and LAMP1 (green). In the yellow box on the right, the upper section displays the enlarged confocal image of the boxed area, and the lower section contains reconstructed 3D images of cells, showing a significant increase in the size and abundance of LAMP1 + puncta consistent with the presence of enlarged lysosomes in the OLs of MPSIIIC mice. **(C)** Representative images of CC of 6-month-old WT and MPS IIIC mice immunolabelled for Olig2 (green) and HS (red). In the yellow box on the right, the upper section displays the enlarged confocal image of the boxed area, and the lower section contains reconstructed 3D images of cells, showing the accumulation of HS in the OLs of MPSIIIC mice. **(D)** Images of CC of 6-month-old WT and MPS IIIC mice immunolabelled for Olig2 (green) and GM3 (red). The yellow box on the left displays the enlarged confocal image of the boxed area, and the right box shows the reconstructed 3D images of the accumulation of GM3 ganglioside in the OLs of MPS IIIC mice. For all panels DAPI (blue) was used as a nuclear counterstain. Bars equal 10 μm. **(E)** TEM micrographs of OLs reveal numerous storage vacuoles, both electrolucent (red arrows) and those containing zebra bodies (yellow arrowheads), as well as swollen mitochondria with largely dissolved cristae (asterisks). High magnification images of boxed areas show a detailed view of storage deposits. Scale bars equal 1 μm. All graphs show individual data, means and SD obtained for three mice per genotype. *P*-values were calculated using an unpaired *t*-test.

To understand the mechanism underlying demyelination, we analyzed the abundance and morphological phenotype of OLs in the CC of MPS IIIC and WT mice. To study oligodendrocyte maturation, we performed co-immunostaining of the brain tissues with antibodies against Olig2, a marker expressed both by oligodendrocytes (OLs) and oligodendrocyte precursor cells (OPCs), and antibodies against CC1, a marker of mature OLs. Our data (Figure 5A and Supplementary Figure 3) showed that the majority of CC1-positive cells in the CC of WT mice were co-labeled for Olig2. On the other hand, in the brains of MPS IIIC mice, we found a significant reduction of CC1-positive, Oligo2-positive as well as Olig2/CC1 double-positive cells suggesting either the reduced production of OLs or their increased degeneration (Figure 5A and Supplementary Figure 3).

The reduced number of OLs coincided with severe morphological changes observed in most of the remaining cells. Specifically, the number and size of LAMP1-positive vacuoles in OLs were increased, consistent with a lysosomal storage phenotype (Figure 5B). Our previous studies revealed that HGSNAT deficiency and impairment of HS catabolism resulted in intralysosomal accumulation of both primary (HS) and secondary (gangliosides) storage materials in neurons and microglia in the brains of MPS IIIC mice (Martins et al., 2015; Pan et al., 2022). To investigate if both compounds are also stored in OLs of MPS IIIC mice, brain tissues were studied by immunohistochemistry using the 10E4 monoclonal antibody, specific for a native HS epitope, and the anti-GM3 ganglioside monoclonal antibody. These experiments revealed increased storage of both HS and GM3 gangliosides in multiple Oligo2-positive cells (Figure 5C and Supplementary Figure 4). TEM analysis further confirmed that OLs in the CC of MPS IIIC mice [identified by the shape and pattern of their nuclei and the presence of multiple microtubular structures in the cytoplasm (Peters and Folger, 2013)] contained multiple storage vacuoles. Some vacuoles exhibited an electrolucent content compatible with storage of HS (Martins et al., 2015; marked with red arrowheads and boxed in Figure 5D), while others contained zebra bodies suggesting storage of myelin fragments or/and sphingolipids (Pagni et al., 2012; marked with yellow arrowheads and boxed in Figure 5D). In addition, OLs in the CC of MPS IIIC mice contained swollen mitochondria with largely dissolved cristae (marked with asterisks in Figure 5E), similar to those we have previously identified in the neurons of MPS IIIC mice (Martins et al., 2015).

### High-field magnetic resonance diffusion imaging reveals microarchitectural changes in the corpus callosum

**FIGURE 6:**
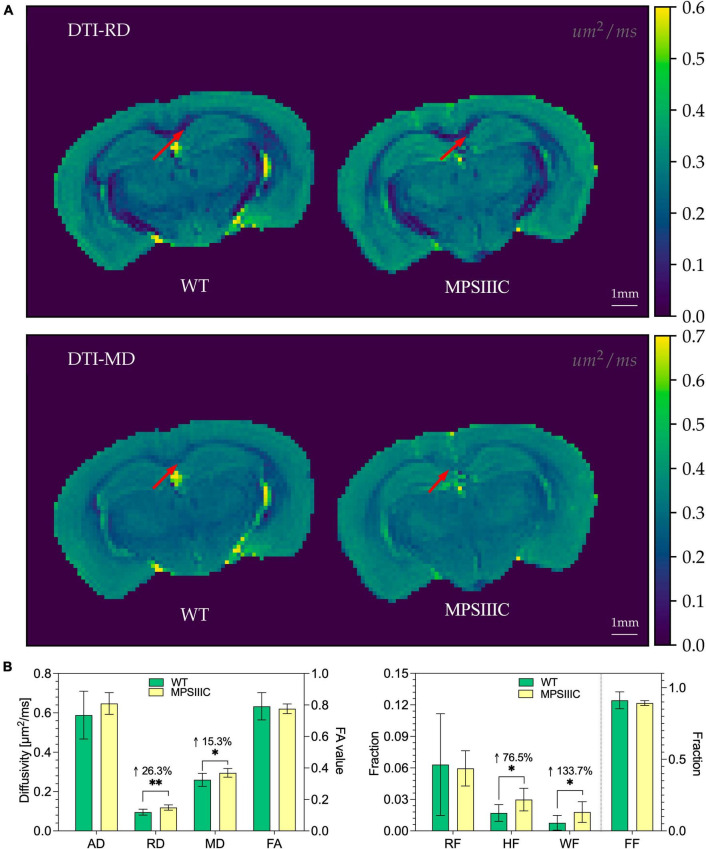
Diffusion maps from DTI and diffusion metrics from DTI and DBSI for WT and MPS IIIC mice. **(A)** Representative images of Radial diffusivity (RD) and Mean diffusivity (MD) maps for brains of WT and MPS IIIC mice demonstrating an increase for both parameters in MPS IIIC brain compared to WT mice. Arrows show the corpus callosum. Diffusivity scale (μm^2^/ms) is shown in the right sidebar. RF, restricted fraction; HF, hindered fraction; WF, water fraction; FF, fiber fraction; AD, axial diffusivity; RD, radial diffusivity; MD, mean diffusivity and FA, fractional anisotropy. **(B)** Bar plots of diffusion metrics from DTI (left) and DBSI (right) of WT and MPS IIIC mice. RD, radial diffusivity; MD, mean diffusivity; Graphs show means ± SD for eight WT (green bars) and 10 MPS IIIC (yellow bars) mice. **P < 0.01, *P < 0.05.

In the corpus callosum, DTI analysis identified clear signs of white matter injury with significant increases in Radial diffusivity (RD, 26%, *P* = 0.003), an indicator of demyelination (Song et al., 2002, 2003), and Mean diffusivity (MD, 15%, *P* = 0.02), a measure known to inversely correlate with white matter maturation (Figure 6). The DBSI analysis showed an increase in Hindered fraction (HF, 76%, *P* < 0.01) and in Water fraction (WF, 134%, *P* < 0.02), that revealed loss of tissue in the CC of MPS IIIC mouse brains compared to controls (Figure 6).

### Myelination defects are pronounced in brain tissues of human MPS III patients

We further analyzed if axonal demyelination was also present in post-mortem tissues collected during autopsy of two MPS IIIC patients whose families provided informed consent for the use of the tissue in this research. The first patient was a 35-year-old Caucasian male with known diagnosis of MPS IIIC. He was wheelchair-dependent since the age of 17 and had the mental capacity of a 2-year-old. The second patient was an 17-year-old female with MPS IIIC diagnosed by biochemical assay of HGSNAT activity at the age of 7 years. The diagnosis was further confirmed by molecular analysis revealing that the patient was homozygous for the c.494-1G > A/p.[P164_S187delinsQSCYVTQAGVRWHHLGSLQALPP GFTPFSYLSLLSSWN, L165PfsX5] mutation affecting the conserved consensus sequence of the splice acceptor site in intron 4 (Martins et al., 2019).

On gross examination, the brain of the first patient showed severe cortical atrophy with fibrotic leptomeninges and enlarged lateral ventricles. The brain of the second patient had a marked decrease in brain weight of 974 g [normal: 1233 ± 115 g (Molina and DiMaio, 2015)] with severe cortical atrophy and enlarged lateral ventricles. However, it did not show any significant leptomeningeal fibrosis. On histological examination, both MPS brains showed neurons with abundant PAS-positive cytoplasmic inclusions (Supplementary Figure 5A) at each of the three levels of the brain examined. These inclusions did not show LFB staining, except for the temporal lobe cortical neurons of the second patient (Supplementary Figure 5B). HES sections of subcortical white matter in MPS brains showed slightly increased cellularity (Supplementary Figures 5C, D) compared to the control brain. The first patient had a focal demyelinating lesion located at the anterior commissure with several hemosiderin-laden macrophages located in proximity (Supplementary Figures 5E–G). LFB staining did not reveal any other regions with profound demyelination.

**FIGURE 7:**
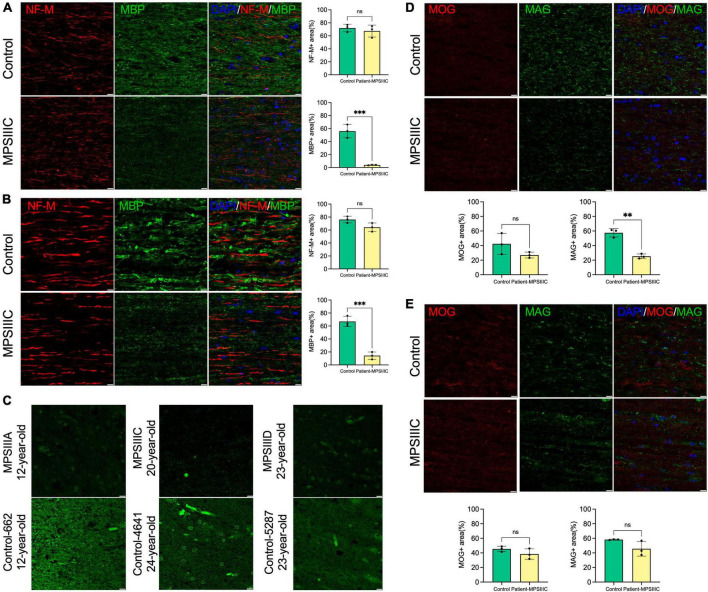
Levels of myelin proteins in the brain and spinal cord of human MPS III patients. **(A,B)** MBP levels are decreased in the brain and spinal cord of human MPS III patients. IHC analysis reveals a significant reduction of MBP + areas (green) on the surface of NF-M + axons (red) in the CC **(A)** and SC **(B)** of 17-year-old MPS IIIC patient compared with age/sex matching control without a neurological disease. Scale bars equal 10 μm. Graphs show quantification of MBP + and NF-M + areas by ImageJ software. Individual results, means and SD of quantification performed in three adjacent areas are shown. *P*-values were calculated using an unpaired *t*-test. **(C)** MBP immunolabelling is also less pronounced in cortex of adult MPS IIIA, MPS IIIC and MPS IIID patients compared with age/sex matching non-MPS controls. Scale bars equal 10 μm. **(D,E)** MAG levels are reduced in the CC but not in the SC of the 17-year-old MPSIIIC patient compared to control, while MOG levels show a non-significant trend toward a decrease. Panels show representative images of CC **(D)** and SC **(E)** of MPS IIIC patient and control stained with MAG + (green) and MOG + (red). Scale bars equal 10 μm. Graphs show quantification of MAG + and MOG + areas by ImageJ software. Individual results, means and SD of quantification performed in three adjacent areas are shown. *P*-values were calculated using an unpaired *t*-test. **P < 0.01; ***P < 0.001; and ns, non significant.

Further examination of the tissues of the second patient by IHC revealed that both CC and the spinal cord (SC) of the patient contained multiple CD68-positive microglia and activated GFAP-positive astrocytes consistent with pronounced neuroinflammation (Supplementary Figure 6). To assess myelination, fixed tissues of CC and SC were examined by IHC using antibodies against MBP (Figures 7A, B), and MAG (Figures 7D, E) which revealed substantially reduced levels of both markers. MOG levels showed a non-significant trend toward a decrease (Figures 7D, E). To determine if the levels of myelin-associated proteins are also reduced in patients affected with other subtypes of MPS III, we analyzed PFA-fixed somatosensory cortex of post-mortem tissue, collected at autopsy and donated to the NIH NeuroBioBank. Samples of 3 MPS III patients (MPS IIIA, MPS IIIC, and MPS IIID) and 3 non-MPS controls, matched for age and sex, were examined (project 1071, MPS Synapse). The age, cause of death, sex, race and available clinical and neuropathological information for the patients and controls are shown in Supplementary Table 2. All MPS patients had complications from their primary disease and died prematurely (before the age of 25 years). None of the patients had received enzyme replacement therapy or hematopoietic stem cell transplantation. This analysis confirmed that the amount of MBP was significantly reduced in cortices from all three MPS patients (Figure 7C), suggesting that demyelination may be a hallmark common for most subtypes of Sanfilippo disease. CC tissues from a 35-year-old MPS IIIC patient did not show any immunoreactivity for MBP or MAG, potentially due to post-mortem changes, which complicated analysis of these markers. We were also unable to achieve immunolabeling of tissues derived from neither 35-year-old nor 17-year-old patient using antibodies against CC1 and Oligo2.

**FIGURE 8:**
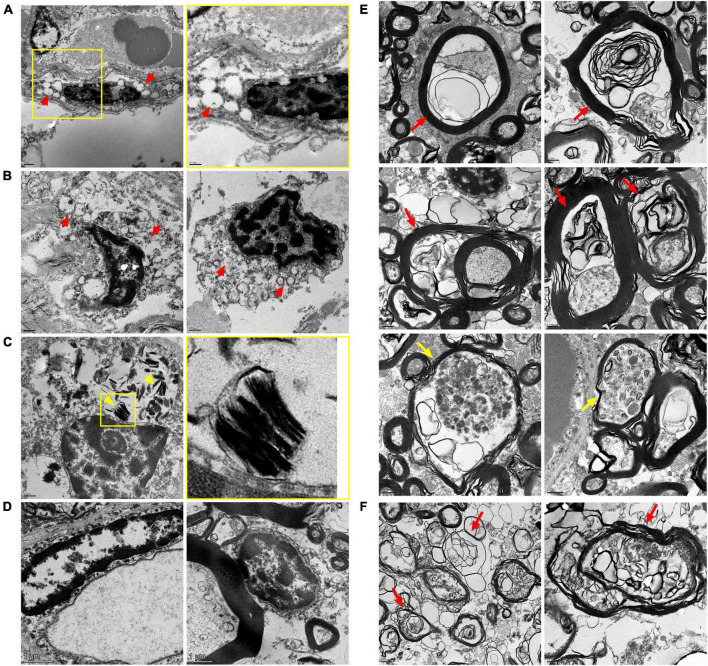
Transmission Electron Microscopy (TEM) analysis confirms microgliosis, pathological changes in oligodendrocyte morphology and axonopathy in the CC of MPS IIIC patients. Electron micrographs show electron-lucent vacuoles (red arrowheads) consistent with HS storage in microglia of the CC of the 17-year-old **(A)** and 35-year-old **(B)** MPS IIIC patients. Oligodendrocytes in the CC of the 17-year-old MPS IIIC patient contain zebra bodies consistent with myelin accumulation [**(C)**, yellow arrowheads]. Microglia (left panel) and oligodendrocytes (right panel) in the CC of control do not contain storage vacuoles **(D)**. Degenerated axons with outfolded and split myelin, sometimes containing cytoplasmic pockets between the sheaths (red arrows), as well as large axonal swellings containing accumulating vesicles (yellow arrows), are observed in the CC of 17-year-old **(E)** and 35-year-old **(F)** MPS IIIC human patients. These structural abnormalities are not observed in the CC of the age and sex matched non-MPS patient.

Despite somewhat poor morphology of CC tissues of 17-year-old and 35-year-old MPS IIIC patients, probably due to their post-mortem provenance, TEM analysis confirmed the presence of multiple microglia containing enlarged electrolucent vacuoles, similar to those present in the microglia in the CC of the MPS IIIC mouse brain, and consistent with storage of HS and other GAGs. OLs in the CC of the 17-year-old MPS IIIC patient also contained electrolucent vacuoles as well as the vacuoles with multilamellar inclusions, consistent with myelin accumulation (Figure 8). Multiple axons in the CC of the patients showed signs of degeneration with outfolded and split myelin containing cytoplasmic materials inside the sheaths. Axonal swellings containing vesicles, similar to those present in the MPS IIIC mice, were also detected. These structural abnormalities were not observed in the CC of the age and sex matched non-MPS patient.

## Discussion

In the current study we identify persistent demyelination as a key component of CNS pathology in MPS IIIC, and likely associated with all diseases of the MPS III spectrum. First, we determined that the amount of MBP, a protein marker of myelinated axons, was drastically reduced in CC of both MPS IIIC mice and MPS IIIC patients at advanced stages of the disease, consistent with myelin disruption and demyelination. Other myelin markers, MAG and MOG, were also reduced. Second, ultrastructural analysis of brain tissue confirmed that the myelin sheaths in the MPS IIIC brains were reduced in thickness and revealed structural abnormalities, including outfolded, empty or split myelin. Third, regions of white matter in MPS IIIC mouse brain were massively infiltrated by activated CD68-positive microglia with lysosomal accumulation of MBP-positive elements and multilamellar fragments (“zebra bodies”), consistent with phagocytosis of myelin fragments. Occurrence of similar processes, associated with demyelination and axonal degeneration, have been reported in multiple white matter pathologies including ischemic injuries, multiple sclerosis and other inflammatory demyelinating diseases, optic nerve diseases, aging, and experimental models of immune encephalomyelitis (Yazdankhah et al., 2021; Alturkustani and Ang, 2022; Correale and Ysrraelit, 2022). In immune encephalomyelitis, activated proinflammatory microglia were reported to directly trigger the loss of myelin, however, in MPS IIIC mice, high levels of microgliosis are observed starting from a very early age (P25 or even before), while the loss of MBP was not detected before the age of 6 months. These findings support the conclusion that axonal demyelination in MPS IIIC is due to aberrant myelin maintenance by resident OLs rather than defects of early axonal development or damage caused by activated microglia or astrocytes. This was supported by our observation that OLs in the CC of MPS IIIC mice were scarce and, in general, immature. Further analysis of OL morphology revealed that they contained multiple electrolucent vacuoles, consistent with storage of HS, and multilamellar bodies, characteristic of lipid accumulation. This was confirmed by IHC, demonstrating that OLs in MPS IIIC but not WT mice were positive for HS and GM3 ganglioside. Based on the presence of these morphological abnormalities, we speculate that the majority OLs in MPS IIIC mice are either dysfunctional or have reduced functionality.

We and others have previously demonstrated that the levels of GM3 ganglioside, together with GM2 ganglioside, lactosylceramide and glucosylceramide, are drastically increased in the brains of mouse models of MPSIII and in the brains of MPS III human patients (Martins et al., 2015; Viana et al., 2020; Pan et al., 2022). The accumulation of these secondary materials mainly occurs in pyramidal neurons in the deep cortex layers and in the CA1-CA3 regions of the hippocampus (McGlynn et al., 2004; Walkley and Vanier, 2009). Our current data show that in contrast to other brain cells, OLs in the CC solely accumulate GM3, while rare GM2-positive cells scattered across the CC are negative for the OL marker Olig2. Although, the mechanism underlying the massive accumulation of GM3 ganglioside in the OLs of MPS IIIC brain requires further investigation, we are tempted to speculate that this phenomenon may be related to the development of myelin defects. Previous studies demonstrated that in NPC the accumulation of GM3 in OLs is a prerequisite for dysfunction and demyelination (Lee et al., 2014). Similarly to MPS III, NP-C manifests with predominant storage of GM2 and GM3 gangliosides (Zervas et al., 2001) and myelin defects in the brain tissues (Maegawa, 2019). In the mouse model of NPC (*Npc1^–/–^* mice) hypomyelination is pronounced in the cerebral cortex and the CC, presumably accounting for the tremor and ataxia observed in these animals (Takikita et al., 2004). The *Npc1^–/–^* mice, heterozygous for the deletion of the *Siat9* (GM3 synthase) gene, showed ameliorated neuropathology, including motor disability and demyelination (Lee et al., 2014). At the same time, deletion of *Siat9* gene resulted in the enhanced neuropathological phenotype and demyelination in *Npc1^–/–^*mice, indicating that the presence (but not an excessive storage) of GM3 ganglioside is essential for OLs development (Lee et al., 2014).

Importantly, OLs in the CC of MPS IIIC mice also exhibited mitochondrial pathology: the majority of mitochondria in these cells had a dysmorphic ballooned appearance with absent cristae. Similar pathology has been previously described by our team in the hippocampal pyramidal neurons of MPS IIIC mice, which was associated with a progressive loss of mitochondrial activity and neurodegeneration (Martins et al., 2015). In the adult brain, once myelination is complete, the long-term integrity of axons is known to depend on the supply of energy to myelinating cells that are key for preserving the connectivity and function of a healthy nervous system (Nave and Werner, 2014). Thus, it is tempting to speculate that mitochondrial dysfunction in OLs, associated perhaps with reduced maturation and increased degeneration, may underlie the demyelination that occurs in the MPS IIIC brain.

Major findings in the mouse MPS IIIC model were confirmed with the brain tissues of human MPS IIIC patients, suggesting that demyelination and white matter injury contribute to the pathology at least at the later stages of the disease. Importantly, LFB staining did not reveal regions with profound demyelination in the brains of both patients, except for one region in the brain of a 35-year-old patient, where the loss of LFB staining was associated with the presence of hemosiderin-laden macrophages suggesting an occurrence of an old micro hemorrhage. This may explain why myelination defects were not previously described in the majority of pathology reports where brain tissues of MPS III patients have been examined using only traditional histochemical techniques such as HES, PAS, and LFB staining. Further studies are required to determine whether patients affected with all subtypes of Sanfilippo disease show a similar degree of demyelination. We consider this to be plausible, as drastically reduced levels of MBP staining were detected in the post-mortem cortical samples of MPS IIIA and MPS IIID patients obtained from NeuroBiobank.

Magnetic resonance diffusion tensor imaging allows generation of quantitative maps that describe tissue microstructure by decomposing diffusion signals into axial diffusivity, radial diffusivity, and fractional anisotropy. We have previously shown how a reduction in radial diffusivity can reliably quantify myelination during development (Lodygensky et al., 2012). Consistent with this, increased radial diffusivity has been used as a biomarker of demyelination (Song et al., 2002, 2003, 2005). A more recent method, Diffusion Basis Spectrum Imaging can provide additional information for an extra-axonal compartment, such as fiber fraction, hindered diffusivity (extracellular), restricted diffusivity (intracellular) and water fraction (Wang et al., 2011). Restricted diffusivity has, in particular, been shown to reflect inflammation with increased cellularity in a mouse model of neuroinflammation (Wang et al., 2014). It has been also applied to detect white matter injury with edema or tissue loss (Chiang et al., 2014; Isaacs et al., 2021; Schiavi et al., 2021). Interestingly, our analysis of diffusivity maps of the CC of 7-month-old MPS IIIC mice revealed a significant increase in radial diffusivity, suggesting loss of myelin together with a strong increase of mid-higher isotropic diffusivity, indicative of edema and tissue loss. In line with the absence of axonal loss, our MRI analysis found no changes in fiber fraction, fractional anisotropy or axial diffusivity. On the other hand, we did not detect significant changes in restricted diffusivity, which was expected considering the high level of microgliosis in the CC of MPS IIIC mice. It is possible, however, that, the model we used was not sufficiently sensitive to detect subtle increase in local cellularity because of the very dense structure of white matter mainly composed of tightly organized fibers.

Together, our data provide novel insights into pathophysiology of Sanfilippo disease. They also identify widespread demyelination in CNS as an important biomarker of disease progression and suggest that analysis of brain myelin by MRI may become in the future a leading non-invasive method for clinical patient assessment.

## Data availability statement

The original contributions presented in the study are included in the article/Supplementary material, further inquiries can be directed to the corresponding author.

## Ethics statement

The studies involving humans were approved by the Research Ethics Board of CHU Ste-Justine. The studies were conducted in accordance with the local legislation and institutional requirements. The participants or their legal representatives provided their written informed consent to participate in this study. The animal study was approved by Animal Care and Use Research Ethics Committee of CHU Ste-Justine.

## Author contributions

MT: Formal analysis, Investigation, Writing – original draft, Writing – review and editing. EZ: Data curation, Formal analysis, Investigation, Writing – original draft. IL: Formal analysis, Investigation, Writing – review and editing. BD: Resources, Supervision, Writing – review and editing. SW: Investigation, Writing – review and editing. JC: Conceptualization, Formal analysis, Supervision, Writing – original draft, Writing – review and editing. TK: Conceptualization, Formal analysis, Methodology, Resources, Supervision, Writing – review and editing. CM: Formal analysis, Methodology, Resources, Supervision, Writing – review and editing. ZC: Formal analysis, Investigation, Methodology, Resources, Writing – original draft, Writing – review and editing. GL: Conceptualization, Data curation, Formal analysis, Funding acquisition, Investigation, Methodology, Resources, Supervision, Writing – original draft, Writing – review and editing. AVP: Conceptualization, Data curation, Formal analysis, Funding acquisition, Methodology, Resources, Supervision, Writing – original draft, Writing – review and editing.
