# Supplementary material for: Severe central nervous system demyelination in Sanfilippo disease
Source: Front Mol Neurosci. 2023 Dec 13;16:1323449. doi: 10.3389/fnmol.2023.1323449 (PMC10756675; doi:10.3389/fnmol.2023.1323449)
Supplement: Supplementary file 1 [file Data_Sheet_1.DOCX]

**Supplementary materials**

**
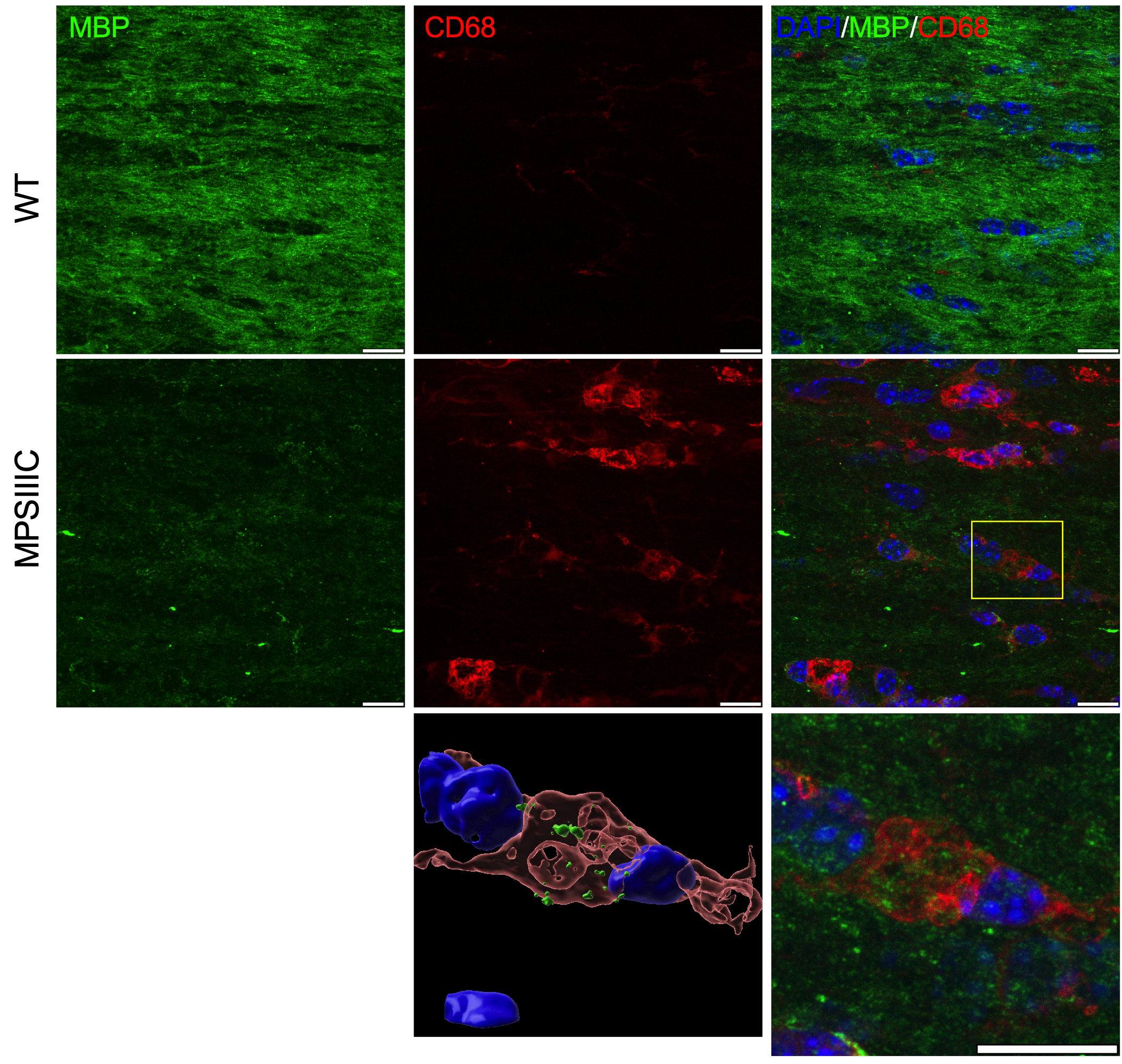
**

**Supplementary figure S1. Intracellular aggregation of MBP in microglia in CC of MPSIIIC mice.**

Panels show representative confocal microscopy images of CC tissue of 6-month-old WT and MPSIIIC mice labelled with antibodies against MBP (green) and CD68 (red). DAPI (blue) was used as a nuclear counterstain. Scale bars equal 10 μm. The enlarged image of the boxed area shows a CD68+ activated microglia containing MBP+ puncta. 3D reconstruction shows that MBP+ puncta are located inside the microglia cell.

**
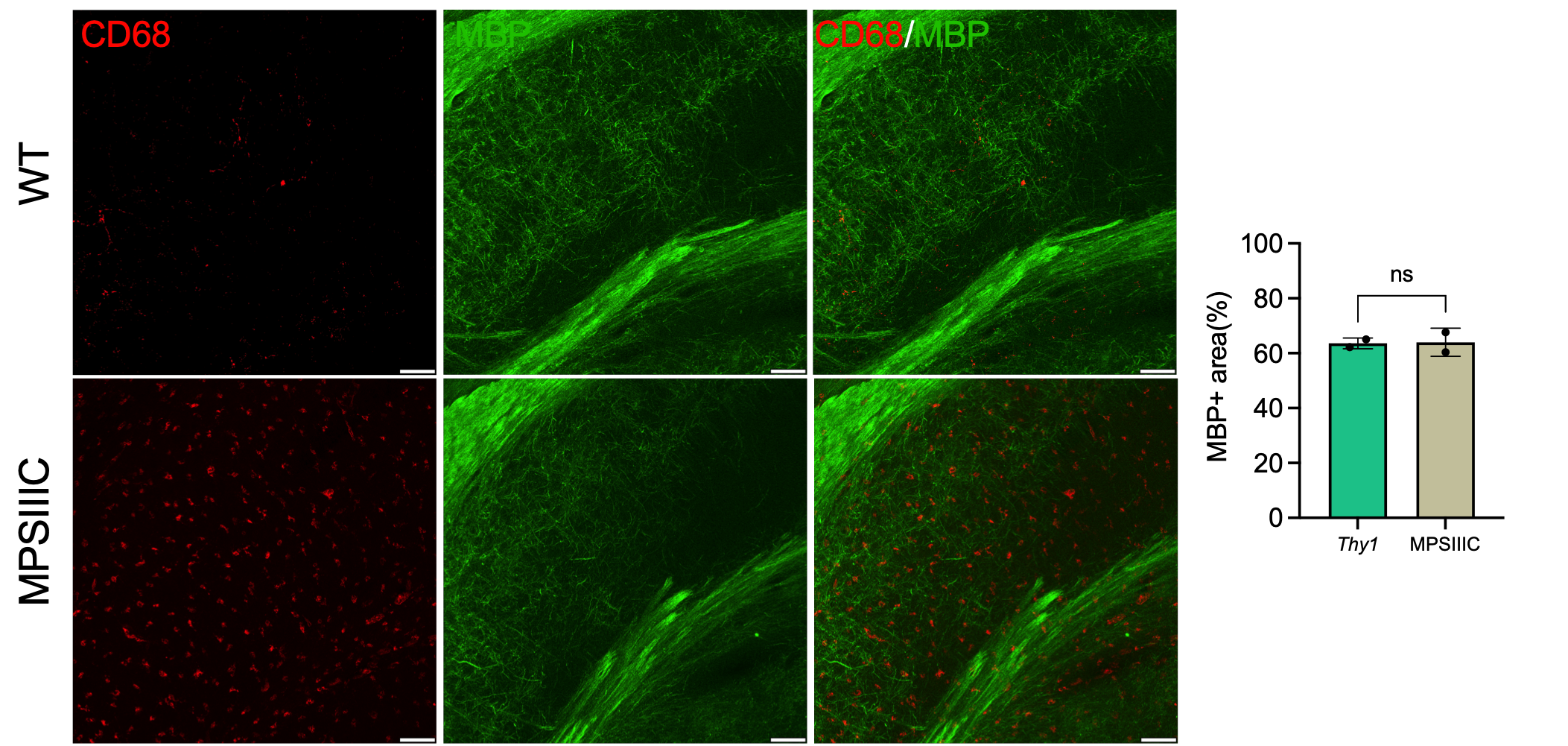
**

**Supplementary figure S2. Microgliosis does not coincide with the loss of myelin at an early age.**

Panels show representative confocal microscopy images of hippocampal and CC tissues of P25 WT and MPSIIIC mice labelled with antibodies against CD68 (red) and MBP (green). Scale bars equal 50 μm.


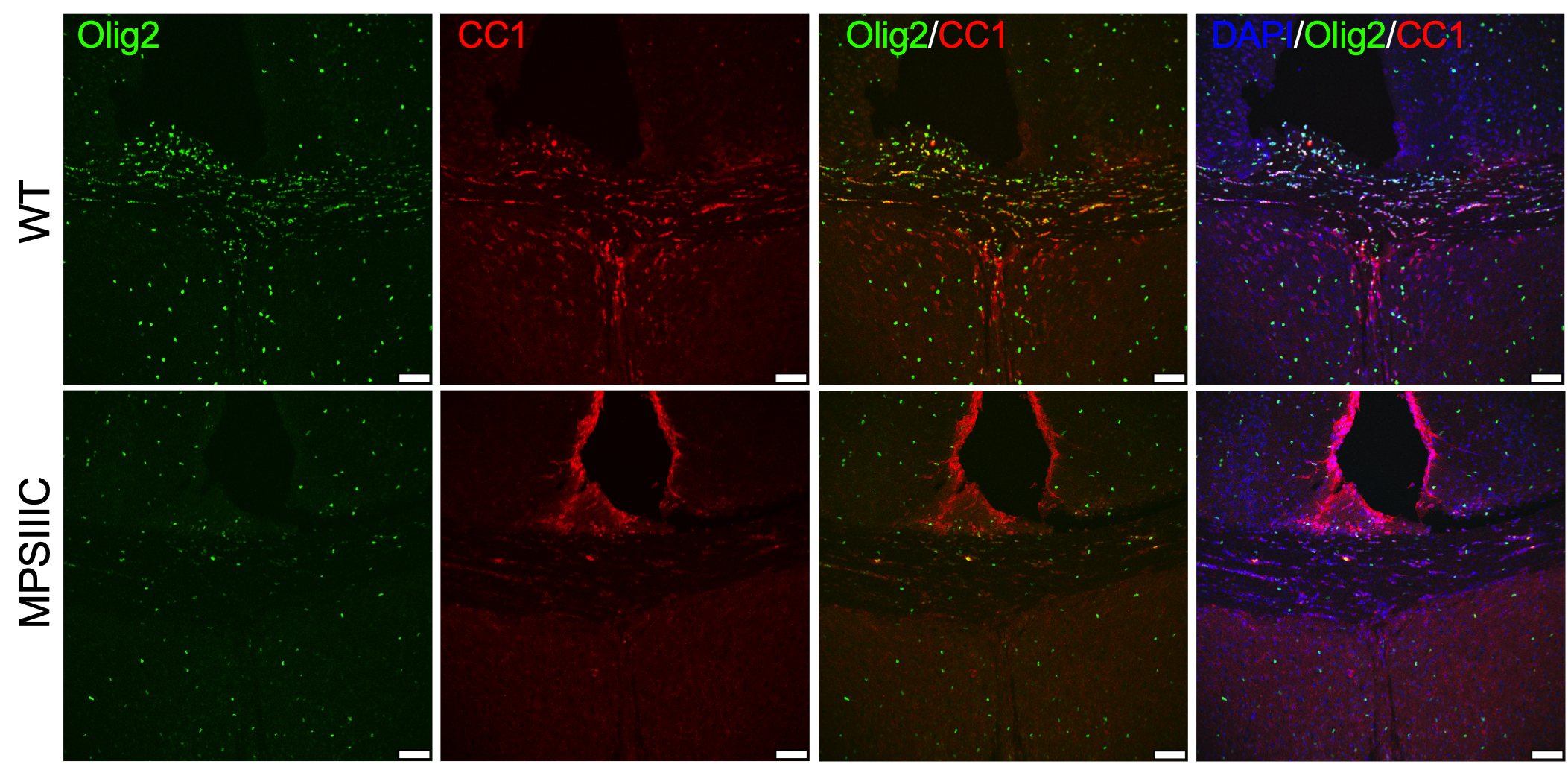


**Supplementary figure S3. CC of MPSIIIC mice show reduced numbers of mature oligodendrocytes.**

Panels show representative images of the CC of 6-month-old WT and MPSIIIC mice immunolabelled for OL lineage marker Olig2 (green) and mature OL marker CC1 (red). DAPI (blue) was used as a nuclear counterstain. Scale bar equals 50 mm.


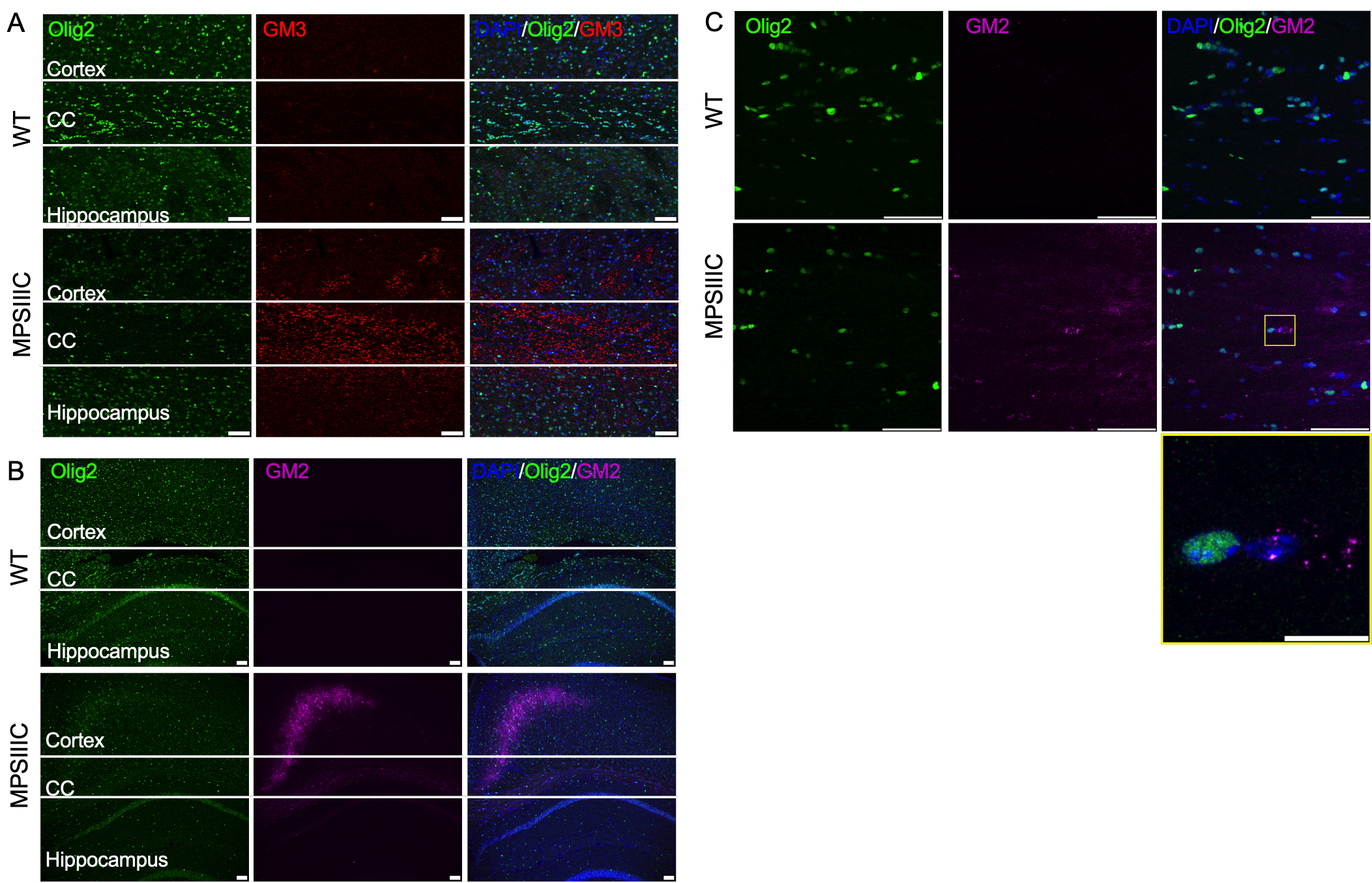


**Supplementary figure S4. Secondary storage of GM3 and GM2 gangliosides in the brain of MPSIIIC mice.**

Panels show confocal microscopy images of the cortex, CC and hippocampus tissues of 6-month-old WT and MPSIIIC mice stained with antibodies against Olig2 (green), GM3 ganglioside (red) **(A)** and GM2 ganglioside (purple) **(B)**, revealing storage of GM3 ganglioside in the CC and GM2 ganglioside in the cortex of MPSIIIC but not of WT mice. **(C)** GM2 ganglioside does not accumulate in OLs in CC tissue of 6-month-old WT and MPSIIIC mice. DAPI (blue) was used as a nuclear counterstain. Scale bar equals 50 mm for A, B, C, and 10 mM for the zoomed image in the panel C.


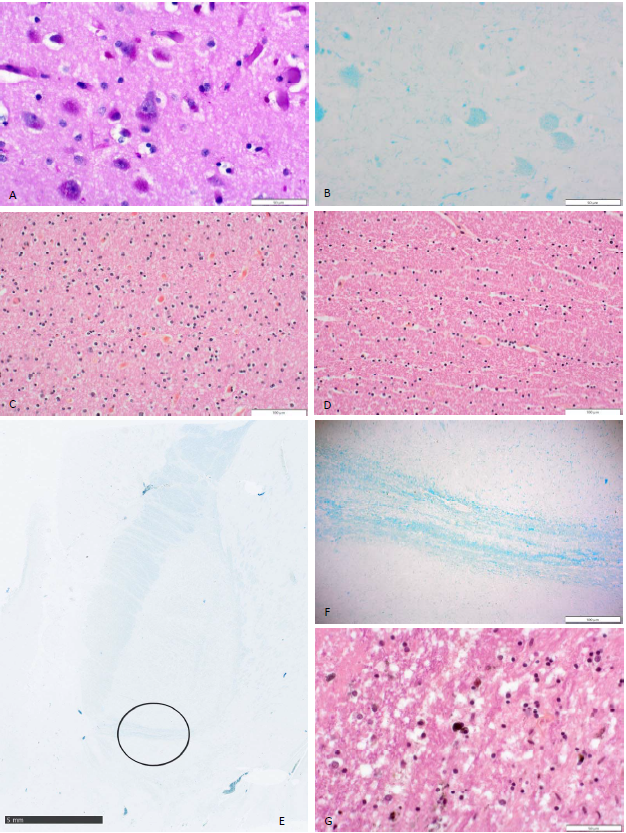


**Supplementary figure S5. Histopathological examination of human brain tissue in MPSIIIC.**

**(A)** PAS-positive neuronal inclusions in patient 1. **(B)** LFB-positive neuronal inclusions in temporal lobe neurons of patient 2. **(C)** Whiter matter hypercellularity in patient 2 compared to control **(D)**. **(E)** Focal demyelinating lesion in the anterior commissure of patient 1, seen in higher magnification in **(F)**. **(G)** Several hemosiderin-laden macrophages are adjacent to the lesion.


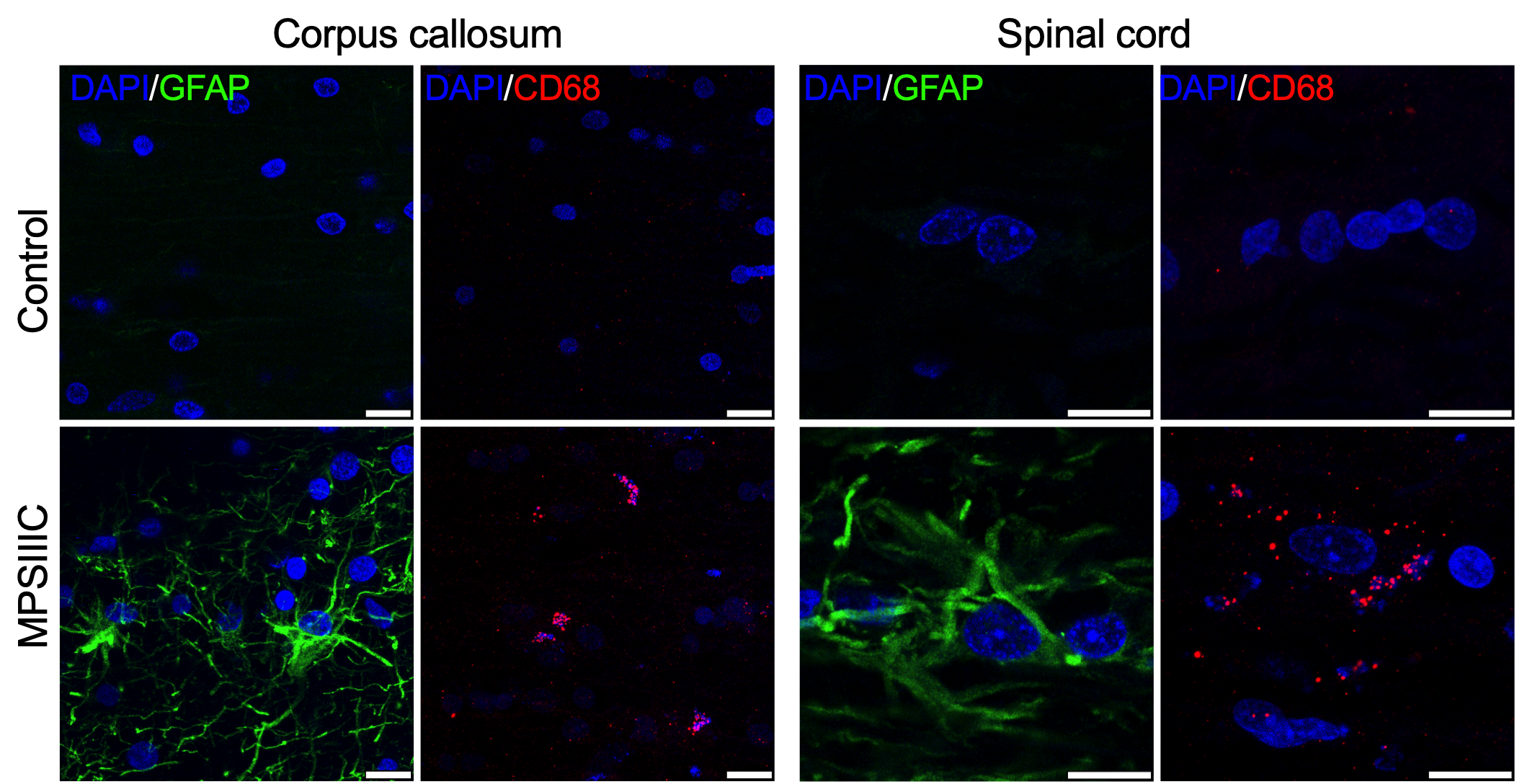


**Supplementary figure S6. Pronounced astromicrogliosis in the corpus callosum and spinal cord of a 17-years-old MPSIIIC patient.**

Multiple GFAP-positive astrocytes (green) and C68-positive microglia (red), indicative of neuroimmune response, are detected in the CC and SC of the MPSIIIC patient but not of the age/sex matching non-MPS control.

**Supplementary Table 1: Antibodies and their dilutions used for immunochemistry**

| **Antigen** | **Host/Target**  **species** | **Dilution** | **Manufacturer** |
| --- | --- | --- | --- |
| APC (CC-1) | Mouse monoclonal | 1:200 | Abcam (ab16794) |
| Olig-2 | Rabbit polyclonal | 1:200 | Sigma-Aldrich (AB9610) |
| GFAP | Rabbit anti-mouse | 1:200 | DSHB (8-1E7-s) |
| CD68 | Rabbit polyclonal | 1:200 | Abcam (ab125212) |
| Heparan sulfate (10E4 epitope) | Mouse anti-mouse | 1:200 | AMSBIO (F58-10E4) |
| Lysosomal-associated membrane protein 1 | Rat anti-mouse | 1: 50 | DSHB (ABL-93-s) |
| Myelin-associated glycoprotein | Mouse monoclonal | 1:200 | Abcam (ab89780) |
| Myelin Basic Protein | Rabbit monoclonal | 1:300 | Abcam (ab218011) |
| Myelin oligodendrocyte glycoprotein | Rabbit monoclonal | 1:500 | Abcam (ab233549) |
| Neurofilament medium chain | mouse anti-mouse | 1:200 | DSHB (2H3-s) |

**Supplementary Table S2: MPS patients and control subjects used in the study**

| 5287 | 4641 | 662 | 5424 | NA | NA | 6194 | 3617 | **UMBN** |
| --- | --- | --- | --- | --- | --- | --- | --- | --- |
| NDAR_INVUB832RTY | NDAR_INVNG087HR2 | NDAR_INVCK582GNX | NDAR_INVUC095YP2 | NA | NA | NA | NDAR_INVFP950EUM | **GUID** |
| Unaffected Control | Unaffected Control | Unaffected Control | MPSIIID, Sanfilippo D Syndrome | MPSIIIC, Sanfilippo C Syndrome | MPSIIIC, Sanfilippo C Syndrome | MPSIIIC, Sanfilippo C Syndrome | MPSIIIA, Sanfilippo A Syndrome | **Disorder** |
| Multiple injuries | Acute asthma | Accident, multiple injuries | Complications of disorder | Complications of disorder | Complications of disorder | Acute pneumonia as a consequence of disorder | Complications of disorder | **Cause of death** |
| 23 | 24 | 12 | 23 | 17 | 35 | 20 | 12 | **Age: years, days** |
| Female | Female | Female | Female | Female | Male | Male | Female | **Sex** |
| Caucasian | African-American | Caucasian | Caucasian | Caucasian | Caucasian | African-American | Caucasian | **Race** |
| NA | NA | NA | One of two siblings suffering from Sanfilippo D, had a progressive decline in hearing, verbal and visual abilities, had no specific cardiopulmonary symptomatology, no seizures, wheelchair bound for the last two years of life. | NA | NA | Had a history of developmental delays, Nissen fundoplication and G-tube, asthma, seizures, sleep problems, agitation, used hearing aids. | NA | **Clinical information** |
| NA | NA | NA | Generalized cerebral atrophy and neuronal storage disorder. | NA | NA | The brain showed cerebral atrophy, mild hydrocephalus, neuronal enlargement with positive cytoplasmic PAS, Alcian blue and LFB, perivascular cuffing of foamy macrophages, white matter vacuolation. | Neurons throughout the brain have enlarged cell bodies with foamy cytoplasm, mild gliosis and status spongiosis in adjacent parenchyma, these neuronal changes are particularly severe in cerebral cortex, Purkinje cell layer of the cerebellum and substantia nigra. The choroid plexus epithelial cells are similarly affected with slightly enlarged and vacuolated cytoplasm. The centrum semi-ovale is mildly gliotic, its perivascular spaces dilated, fibrotic and contain glitter cells and occasional lymphocytes. | **Neuropathologic findings** |
